# Similar Sensorimotor Activations with and without Virtual Limbs During Action Execution and Observation in Neurorehabilitation Systems

**DOI:** 10.1007/s10548-026-01219-1

**Published:** 2026-05-27

**Authors:** Cristián Modroño, Sergi Bermúdez-Badia, M S Cameirão, F Marcano-Serrano, F Pereira, T Paulino, E Hernández-Martín, B R García-Ramos, R Villarroel, J Plata-Bello, J L González-Mora

**Affiliations:** 1 Departamento de Ciencias Médicas Básicas, Universidad de La Laguna, Tenerife, Spain; 2 Instituto de Tecnologías Biomédicas, Universidad de la Laguna, Tenerife, Spain; 3 Instituto Universitario de Neurociencia, Universidad de la Laguna, Tenerife, Spain; 4 ARDITI - Agência Regional para o Desenvolvimento de Investigação, Tecnologia e Inovação, Funchal, Portugal; 5 Faculdade de Ciências Exatas e da Engenharia & NOVA LINCS, Universidade da Madeira, Funchal, Portugal; 6 Departamento de Psicología Evolutiva y de la Educación, Universidad de La Laguna, Tenerife, Spain; 7 Departamento de Neurocirugía, Hospital Universitario de Canarias, Tenerife, Spain; 8 Instituto de Investigación Sanitaria de Canarias (IISC), Tenerife, Spain

**Keywords:** Mirror neuron system, Virtual limbs, fMRI, Action observation, Neurorehabilitation

## Abstract

Virtual reality rehabilitation systems often represent actions through virtual limbs. However, previous observation-only studies suggest that frontoparietal networks involved in action observation and execution may also be engaged by simpler virtual effectors. In the present fMRI study, we examined whether the visual presence of a virtual limb modulates brain activity during both action execution and action observation in a virtual neurorehabilitation task. Twenty-four healthy right-handed volunteers performed a cancellation task under four conditions: dot execution, hand execution, dot observation, and hand observation. Analyses showed similar frontoparietal and sensorimotor activations across hand and dot conditions in both runs, and direct hand-versus-dot comparisons did not reveal significant corrected differences. Under the visuomotor conditions tested here, the visual presence of a virtual limb was not associated with substantial differences in overall frontoparietal/sensorimotor activations. These findings support the development of more flexible virtual rehabilitation tasks and broaden the range of visual effectors that may be considered in future system design.

**Supplementary Information:**

The online version contains supplementary material available at 10.1007/s10548-026-01219-1.

## Introduction

Mirror neurons are activated not only when an individual performs a specific action (such as reaching for food) but also when they watch others perform the same or a comparable action (Dipellegrino et al. 1992; Gallese et al. 1996, 2002). In this way, the neuron reflects the actions of another, as if the observer were performing the action themselves. Mirror neurons were first discovered in monkeys using electrodes placed in the premotor and parietal cortices. Later studies in humans, utilizing non-invasive neuroimaging and neurophysiological techniques, provided evidence of a frontoparietal cortical network with similar properties. This network, known as the Mirror Neuron System (MNS), is believed to play a significant role in both understanding actions and facilitating imitation (Cattaneo and Rizzolatti 2009). In humans, however, this question is typically addressed at the systems level rather than at the level of individual neurons. Because fMRI does not allow inferences about individual neuronal responses, we adopt a network-level perspective. Accordingly, the term MNS is used here as a system-level shorthand for frontoparietal regions implicated in the observation and execution of actions, rather than as a claim about the response properties of individual neurons.

The fundamental characteristic of mirror neurons (namely, their activation during both performed and observed actions) has inspired therapeutic approaches in neurorehabilitation, particularly action observation therapy. This approach involves visually presenting actions to engage motor-related frontoparietal networks, which can aid in the reorganization of brain motor regions affected by a stroke (Bhasin et al. 2012; Boni et al. 2023; Borges et al. 2018; Buccino et al. 2006; Garrison et al. 2010; Robinson-Bert and Woods 2024; Sale and Franceschini 2012) and can be helpful for patients with difficulties to perform active movements. During therapy, patients are typically asked to observe various motor actions, such as arm-reaching movements or object-related motor tasks (Harmsen et al. 2015; Seitz et al. 2024). When feasible, patients may also be asked to execute these actions (the capability to execute such actions depends on the affected brain regions and the stage of recovery). Several recent quantitative reviews of randomized controlled trials (Borges et al. 2018; Peng et al. 2019; Zhang et al. 2023) have shown that this therapy may be beneficial to improve upper limb motor function in stroke patients.

Action observation therapy often involves watching other people perform motor actions through video or in real time (Borges et al. 2018). However, it can also be based on virtual reality (VR) techniques, which have gained prominence in recent years as valuable tools for sensorimotor neurorehabilitation. VR systems enhance patient motivation, provide specific feedback, increase training volume, and allow for calibrated difficulty levels (Bermúdez i Badia et al. 2016; Fluet et al. 2022; Villarroel et al. 2025). Consequently, many neurorehabilitation systems and experimental tasks have been developed based on observing or controlling a virtual limb performing various actions with virtual objects (Alves et al. 2016; Ballester et al. 2015; Holper et al. 2010; Kang et al. 2012; Vourvopoulos & Badia, 2016). For instance, one neurorehabilitation system presents patients with virtual objects that approach and are intercepted by virtual arms (Cameirao et al. 2007, 2010), thereby engaging frontoparietal regions (Prochnow et al. 2013). Observing intransitive movements (those not involving interaction with objects), such as virtual limb flexions, extensions, or sequences of finger movements, has also been employed in this approach (Adamovich et al. 2009; Boos et al. 2011; Tunik et al. 2013). Moreover, research has shown that the activity elicited is similar for both real and virtual stimuli (Brihmat et al. 2018).

Interestingly, it may not be necessary to represent limbs in virtual rehabilitation systems. In an exploratory experiment (Modroño et al. 2013), significant frontoparietal activity was obtained by simply watching a virtual object’s movements, such as a paddle (the reason for this activity is that the movements of the virtual object were likely perceived as motor acts familiar to the observer, and this familiarity is a prerequisite for substantial activation in the parietofrontal MNS (Buccino et al. 2004; Calvo-Merino et al. 2005)). Expanding on this idea, a virtual neurorehabilitation system study (Modroño et al. 2019) revealed similar activity with or without displaying a virtual arm. However, these results were obtained under observation-only conditions with a small sample size.

Since action observation therapy typically includes execution tasks when feasible, the present study focuses on both observation and execution in neurorehabilitation tasks, with and without a virtual limb. We conducted an fMRI experiment with a larger sample of twenty-four healthy volunteers who performed action observation and action execution tasks. We hypothesize that similar activations will occur regardless of the virtual limb’s presence. If confirmed, this finding could have significant implications for designing virtual rehabilitation systems, expanding the use of tasks that do not require virtual limbs and offering greater flexibility in system development.

## Methods

### Participants

Twenty-four right-handed, neurologically healthy subjects (10 female, 14 male; mean age = 22.4 years, SD = 2.2) participated in the experiment. They were recruited from university students and had normal or corrected-to-normal vision. The study was approved by the local Ethics Committee (University of La Laguna) and conducted in accordance with the Declaration of Helsinki.

### Neurorehabilitation Task

**Fig. 1:**
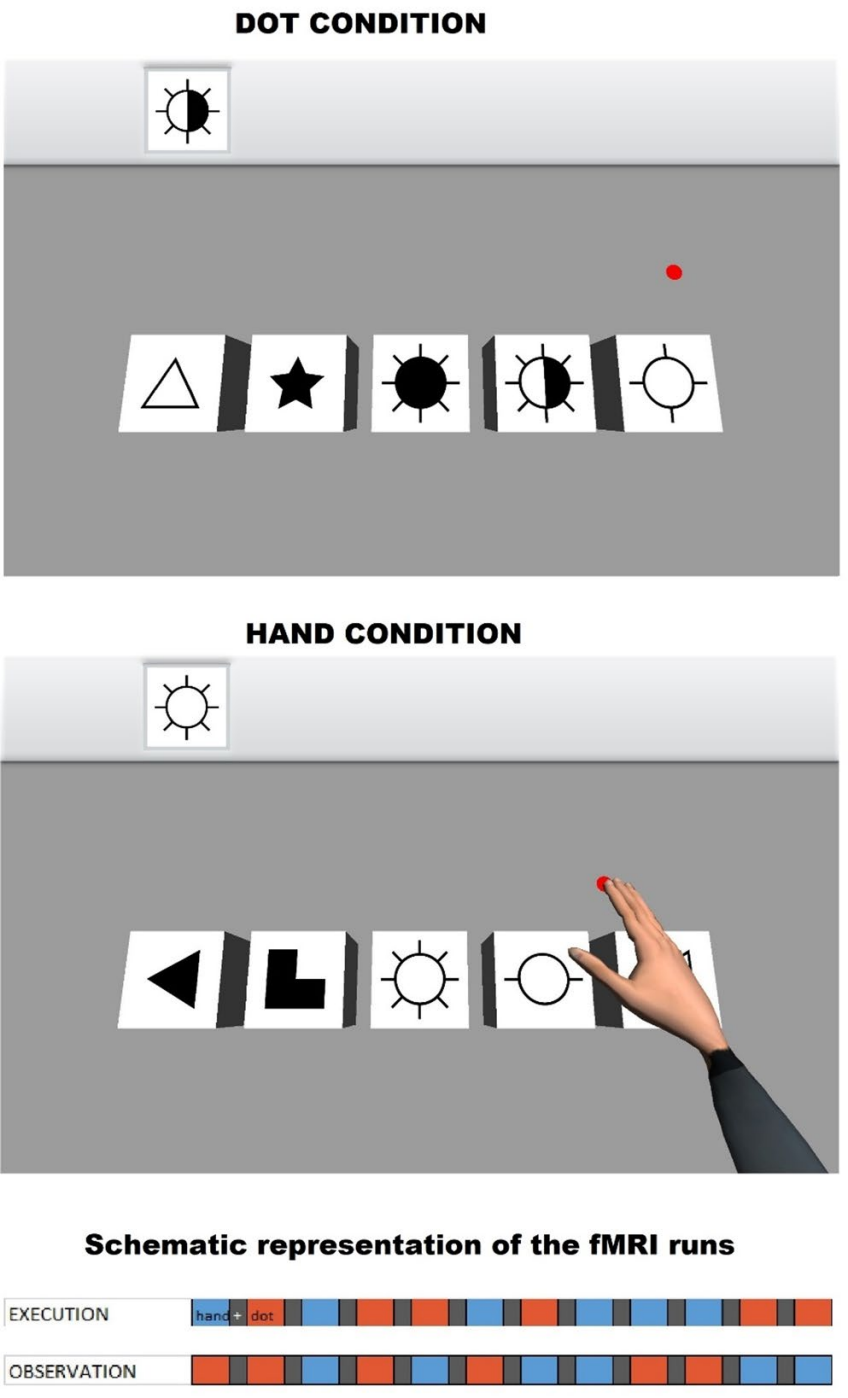
The cancellation task. During the execution run, participants controlled either a red dot alone (dot condition) or a virtual limb with a red dot positioned under the tip of the middle finger (hand condition). In both conditions, participants were required to select the figure indicated in the top-left corner of the screen. Since the experiment did not focus on cognitive aspects such as memory, the target figure remained visible throughout each trial to minimize cognitive load. During the observation run, the cancellation task was performed automatically by the system while participants remained still, observing the displayed actions. BOTTOM: Schematic representation of the runs. Both runs consisted of hand, dot, and fixation blocks, represented in blue, red, and gray, respectively

Participants engaged in a neurorehabilitation task, illustrated in Fig. 1. This task was a simplified version of Reh@Task (Faria et al. 2016), a virtual reality system that combines arm reaching, attention and memory training. One of the advantages of Reh@Task is its flexibility, as it can be easily customized to test different research hypotheses related to sensorimotor or cognitive aspects. For the present experiment, the sensorimotor component of Reh@Task was of greater interest than its cognitive component. The customized version used in this study presented a cancellation task in which participants identified a single abstract symbol (the target) among four distractors in a three-dimensional environment. An icon showing the target symbol was always displayed in the top-left corner of the screen during each trial (Fig. 1). In this way, cognitive demands were minimized, as the task did not require significant memory or sustained attention, thereby maintaining a low cognitive load. The task required moving a virtual cursor and positioning it over the target element for 1.5 s. During the task, participants interacted with either: (1) A virtual hand and arm with a red dot positioned under the tip of the middle finger, or (2) A red dot alone to perform the selection process. Skeletal constraints using inverse kinematics ensured the movements of the virtual hand were physically accurate and plausible. Since all participants were right-handed, a virtual right arm was displayed for all. The movements of the effector (dot/hand) started from the right side of the display (consistent with the use of a right virtual arm) and proceeded toward the cued target, as illustrated in Fig. 1. Upon completion of each trial, the process restarted with a new randomly selected target and distractor elements. Depending on the experimental condition (described below), participants either used a joystick with their right hand to complete the task or observed the task being completed automatically by the system.

### Data Acquisition

A simplified version of Reh@Task was executed on a PC located in the MRI control room. This PC also ran LabChart (ADInstruments, Dunedin, New Zealand), a physiological data analysis software that integrates multiple recording devices. LabChart received input signals marking the precise timing of fMRI pulse sequences via the PowerLab data acquisition hardware (ADInstruments, Dunedin, New Zealand). Simultaneously, it recorded event markers from Reh@Task, which had been previously modified by its developers to support this communication by sending event markers to PowerLab through an Arduino board. LabChart logs were later processed offline using a custom MATLAB script to extract the onset times and durations of each participant’s trials.

The experiment consisted of two parts, each corresponding to one fMRI run. (1) Execution Run: Participants performed the cancellation task using their right hand and an MRI-compatible joystick (Resonance Technology Inc., Northridge, CA) to control the virtual dot/hand. (2) Observation Run: The system automatically completed the cancellation task while participants remained still, observing the displayed actions. Participants were instructed to minimize head and trunk movement during both runs and to focus on the movements of the dot and the hand during the observation run. Although eye tracking was not recorded, the visual characteristics of the task were such that attention was naturally directed toward the effector and its movement toward the target. Visual stimuli were delivered via MRI-compatible eyeglasses (Visuastim, Resonance Technology, Northridge, CA) with a resolution of 800 × 600 pixels, 32-bit color depth, and a refresh rate of 60 Hz. The visual field covered an angle of 30 × 22.5°.

The execution run consisted of three conditions: dot, hand, and fixation. Dot Condition: Participants controlled the movement of the dot during six blocks of 55 s each (up to 12 cancellation trials per block, depending on how quickly participants completed each trial; see also Results section). Each new trial started immediately after completion of the previous one. Hand Condition: Similar to the dot condition, but a virtual limb appeared above the dot (Fig. 1). Fixation Condition: Participants fixated on a gray cross in the center of a black screen (baseline) for 21 s. The dot and hand blocks were presented in random order and separated by fixation blocks (Fig. 1, bottom). The block sequence was identical for all participants. Before the execution run, participants completed a five-minute practice session inside the MRI scanner to familiarize themselves with the task. The observation run was structured similarly to the execution run, but participants passively observed the cancellation task being completed automatically by the system.

For each run, 445 axially oriented functional images were obtained by a 3T Signa HD MR scanner (GE Healthcare, Milwaukee, WI) using an echo-planar-imaging T2*-weighted gradient-echo sequence and an 8-channel head coil. The scanning parameters were: TR = 2000 msec, TE = 21.6 msec, flip angle = 75º, matrix size = 64 × 64 pixels, 36 slices, 4 × 4 mm in plane resolution, spacing = 4 mm, slice thickness = 3.3 mm, interleaved acquisition. Slices were aligned with the anterior commissure-posterior commissure (AC-PC) line and covered the entire brain. Functional scanning began after 10 s of dummy scans to ensure tissue steady-state magnetization. High-resolution anatomical T1 images were also acquired for anatomical reference using a 3D fast spoiled-gradient-recalled pulse sequence with the following parameters: TR = 8.84 msec, TE = 1.75 msec, FA = 10º, matrix size = 256 × 256 pixels, 1 × 1 mm in plane resolution, between slices = 1 mm, slice thickness = 1 mm.

### Data Analysis

The imaging data were checked for artifacts before being preprocessed and analyzed using SPM12 software (www.fil.ion.ucl.ac.uk/spm/). Raw functional images were realigned to their mean image and unwarped (correction of susceptibility-induced distortions) to reduce residual head motion-related variance (Andersson et al. 2001). The images were then coregistered to the anatomical T1 and normalized to the MNI space using the unified normalization segmentation procedure, with normalization success validated by visual inspection. The normalized images (2 × 2 × 2 mm resolution) were smoothed using an 8 × 8 × 8 mm Gaussian kernel at full width at half maximum (FWHM).

A block design within the framework of a general linear model (GLM) was employed for individual subject analyses (first level) to investigate differences in brain activity during the *dot*,* hand*, and *fixation (fix)* conditions. The first-level design matrix included two sessions (*execution* and *observation* runs), each with three conditions (*dot*,* hand*, and *fixation*), resulting in six modeled conditions: *dot exe*,* hand exe*,* fix exe*,* dot obs*,* hand obs*,* and fix obs*. The onsets and durations of the blocks were obtained from the LabChart logs, as described in the data acquisition subsection. Each block, rather than each individual trial within the block, was modeled using a boxcar function convolved with the hemodynamic response function (HRF). This block-based approach was chosen because the trials within each block involved the same task structure and because the main aim was to compare sustained condition-related activity across the experimental conditions. A temporal high-pass filter (128 s) was applied to remove slow signal drifts. Activation maps for each participant were generated by applying t-statistics, and four pairwise contrasts of interest were computed at the first level: dot *exe > fix exe*,* hand exe > fix exe*,* dot obs > fix obs*, and *hand obs > fix obs.* These contrasts were used to compare brain activation in the dot and hand conditions relative to the fixation baseline, separately for the execution and observation runs.

First-level contrast images were used for random-effects group analysis (second level analysis). This analysis was conducted using a 2 × 2 full factorial design with the factors Effector (*dot*,* hand*) and Run (*execution*,* observation*). SPM t-contrasts were applied to the ANOVA parameter estimates to characterize brain regions activated during the experimental conditions. The factorial model was also used to examine the main effects of Run and Effector and their interaction. Anatomical locations and Brodmann areas were identified using the xjView 10.0 toolbox (www.alivelearn.net/xjview/). Statistical maps were thresholded at *p* < 0.05, false discovery rate (FDR) corrected at the voxel level for multiple comparisons, with a minimum cluster size (k) of 25 voxels.

After the group analysis, three conjunction analyses were performed using the Minimum Statistic compared to the Conjunction Null method (Nichols et al. 2005) to identify regions commonly activated during both action observation and action execution. The conjunctions included: (1) Dot conditions: (*dot obs* ∩ *dot exe*) (2) Hand conditions: (*hand obs* ∩ *hand exe*) (3) Dot and hand conditions: (*dot obs* ∩ *dot exe* ∩ *hand obs* ∩ *hand exe*). For these analyses, group contrast statistical activations were thresholded, binarized, and multiplied using the same voxel-level FDR-corrected threshold described above. This procedure was implemented as a post-processing step to visualize the spatial overlap between observation and execution contrasts, resulting in conjunction maps showing regions commonly activated during both tasks (Fig. 3).

In addition, we performed a priori ROI analyses in anatomically defined frontoparietal and sensorimotor regions using WFU PickAtlas and MarsBaR. The ROI set included bilateral inferior frontal gyrus (BA44/45), premotor cortex (BA6), primary motor cortex (BA4), inferior parietal lobule (BA40), superior parietal lobule (BA7), and supplementary motor area. Within WFU PickAtlas, all regions except the SMA were derived from the TD Brodmann Areas+ atlas, whereas the SMA ROI, which is not included in that atlas, was defined using the corresponding region from the JuBrain Anatomy Toolbox. Mean contrast estimates were extracted for each subject and condition, and hand-versus-dot comparisons were performed separately for execution and observation. For each ROI comparison, we report the mean difference (hand − dot), 95% confidence intervals, and Cohen’s dz. P values were corrected for multiple comparisons across ROIs within each run using the Bonferroni procedure.

Finally, as a descriptive performance measure, we compared the mean number of completed trials per block between the hand and dot conditions during execution. This measure did not differ significantly between conditions (hand: M = 8.55 trials/block; dot: M = 8.65 trials/block; mean difference [hand − dot] = − 0.10, 95% CI [− 0.38, 0.17], *p* = 0.437).

## Results

**Fig. 2:**
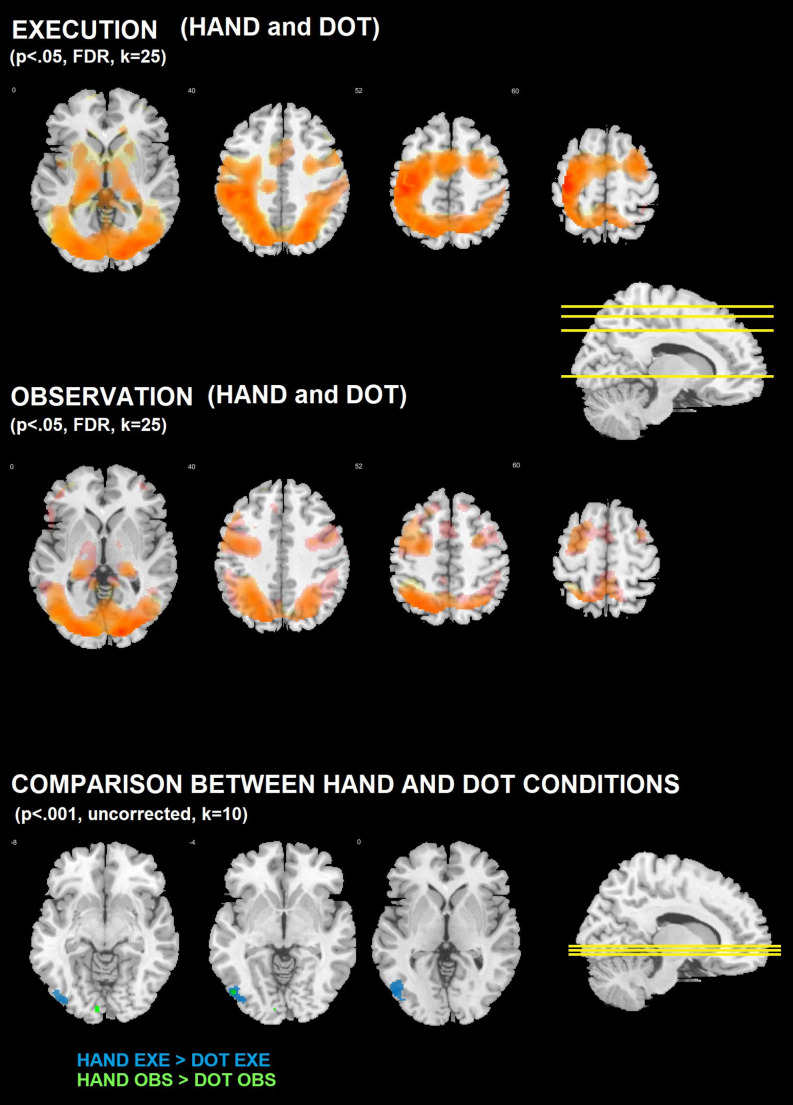
Brain activations during the dot and hand conditions in the observation and execution tasks were similar (the small numbers near the slices indicate the Z-coordinate in MNI space). *TOP: Execution task.* The execution task was associated with activations in frontoparietal and other sensorimotor regions, such as the left primary motor cortex, during both conditions: when the virtual hand was present (*hand exe > fix exe*; red voxels) and when it was absent (*dot exe > fix exe*; yellow voxels). Regions activated in both conditions are shown in orange. Notably, the same sensorimotor regions were activated in both execution conditions, which explains why most regions appear in orange rather than red or yellow (*p* < 0.05, FDR corrected at the voxel level; k = 25). The contrasts *hand exe > dot exe* and *dot exe > hand exe* did not show significant results at this significance level. *MIDDLE: Observation task.* The observation task was associated with activations in frontoparietal regions commonly involved in action observation and execution, both when the virtual hand was moving (yellow voxels; *hand obs > fix obs*) and when only the red dot was moving (red voxels; *dot obs > fix obs*). Regions activated in both conditions are shown in orange. Similar sensorimotor regions were activated in both observation conditions, which explains why most areas appear in orange rather than red or yellow (*p* < 0.05, FDR corrected at the voxel level; k = 25). The contrasts *hand obs > dot obs* and *dot obs > hand obs* did not show significant results at this significance level. *BOTTOM: Comparison between hand and dot conditions (uncorrected statistics).* At a less conservative threshold (*p* < 0.001, uncorrected, k = 10), the contrasts *hand obs > dot obs* and *hand exe > dot exe* showed small clusters of increased activity in occipital regions associated with visual processing. The symmetric contrasts (*dot obs > hand obs* and *dot exe > hand exe*) did not yield significant results at this threshold

Figure 2; Table 1 illustrate the brain regions activated during the execution and observation tasks under the dot and hand conditions. The observation task elicited increased activity in frontoparietal regions commonly associated with action observation and execution, including the parietal lobe, premotor cortex, and the caudal part of the inferior frontal gyrus (Cattaneo and Rizzolatti 2009). This increase was observed not only when the virtual hand was present but also when only the moving dot appeared on the screen. Additionally, other visuomotor regions, such as the occipital lobe and cerebellum, were activated in both conditions.

Interestingly, no significant differences in brain activity were detected between the hand and dot conditions (*hand obs > dot obs* and *dot obs > hand obs* contrasts; *p* < 0.05, FDR-corrected for multiple comparisons at the voxel level, with a minimum cluster size [k] of 25 voxels). When using a less conservative threshold (*p* < 0.001, uncorrected, k = 10), small clusters of increased activity appeared in visual processing areas of the occipital regions for the *hand obs > dot obs* contrast (Fig. 2). However, no activations were found in the sensorimotor systems.

For the execution task, brain activations were also similar regardless of whether the hand was present. These activations were observed in both cortical and subcortical regions typically involved in visuomotor tasks, including frontoparietal regions commonly associated with action observation and execution. Regions such as the supplementary motor area, premotor cortex, primary motor cortex, basal ganglia, thalamus, parieto-occipital regions, and cerebellum were activated (Hardwick et al. 2018; Modroño et al. 2020). Activations were found in the left (but not in the right) primary motor cortex, consistent with the task being performed using the right hand. As with the observation task, no significant differences in brain activity were found between the hand and dot conditions (*hand exe > dot exe* and *dot exe > hand exe* contrasts; *p* < 0.05, FDR-corrected at the voxel level, k = 25). At the less conservative threshold (*p* < 0.001, uncorrected, k = 10), small clusters of increased activity were observed in the occipital regions for the *hand exe > dot exe* contrast (Fig. 2). Again, no activations were detected in the sensorimotor systems.

In addition to the simple condition-versus-fixation contrasts, we examined the main effects of Run and Effector, as well as their interaction, within the 2 × 2 factorial design. The main effect of Run (execution > observation) showed the expected predominance of motor-related activations during execution. The inverse contrast (observation > execution) showed a more limited and heterogeneous pattern of activations, involving temporomedial, temporoparietal, insular, and medial frontal regions. The main effect of Effector was mainly associated with visual-occipital differences, whereas the Effector × Run interaction did not reveal a clear pattern of robust effects. For completeness, these analyses are reported in Supplementary Figures S1, S2, and S3.

A priori ROI analyses did not reveal hand–dot differences that survived correction for multiple comparisons in any of the predefined regions (Supplementary Table 1). During execution, the differences between hand and dot were small across all ROIs, with confidence intervals including zero in every case. During observation, nominally significant uncorrected effects were observed only in bilateral IFG (left IFG: hand − dot = − 0.142, 95% CI [− 0.263, − 0.021], *p* = 0.024, dz = − 0.49; right IFG: hand − dot = − 0.133, 95% CI [− 0.265, − 0.001], *p* = 0.049, dz = − 0.42), but these effects did not survive correction for multiple comparisons and were therefore treated as exploratory.

**Fig. 3:**
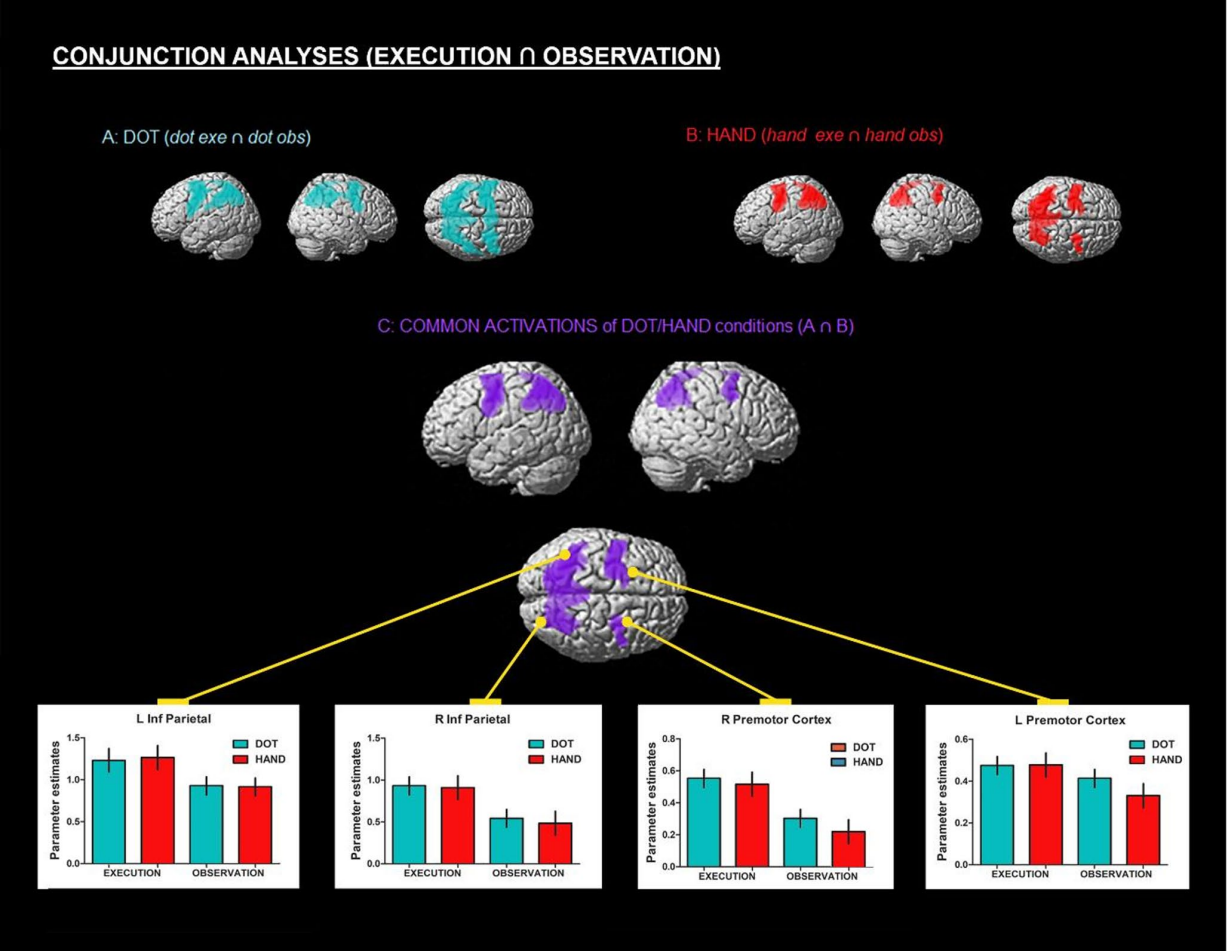
Three conjunction analyses. Results of conjunction analyses showing frontoparietal regions bilaterally activated during both execution and observation of the virtual rehabilitation task. These results highlight similar regions for (**A**) dot and (**B**) hand conditions (*p* < 0.05, FDR corrected at the voxel level; k = 25). Activation maps were inclusively masked with the frontal and parietal lobes to focus on frontoparietal regions commonly activated during action observation and execution. (**A**) Conjunction of the dot conditions (*dot exe* ∩ *dot obs*). (**B**) Conjunction of the hand conditions (*hand exe* ∩ *hand obs*). (**C**) Conjunction of the dot and hand conditions (*dot exe* ∩ *dot obs* ∩ *hand exe* ∩ *hand obs*). Parameter estimates for each of the conditions (*dot exe*,* dot obs*,* hand exe*, and *hand obs*) are shown for four representative voxels: left inferior parietal lobule [-36 -56 54], right inferior parietal lobule [32–60 46], left premotor cortex [-38 -6 44], right premotor cortex [28 0 48]; error bars depict the standard error

Figure 3 presents the results of the three conjunction analyses performed to identify regions activated by both the execution and observation of the virtual task. The first conjunction analysis was limited to the dot conditions, the second to the hand conditions, and the third encompassed both dot and hand conditions. Activation maps were inclusively masked with frontal and parietal lobes to restrict the analysis to the frontoparietal cortex. All three analyses revealed similar activations within frontoparietal regions commonly activated during action observation and execution (*p* < 0.05, FDR-corrected at the voxel level, k = 25) (although the dot conjunction appeared somewhat more extensive than the hand conjunction in some regions, no significant corrected differences were found in the direct hand-versus-dot comparisons reported above; therefore, this pattern does not alter the main interpretation of the results). Bilateral activations were observed around the premotor cortex and the caudal part of the frontal gyrus (precentral gyrus and caudal parts of the superior, middle, and inferior frontal gyri). Additionally, bilateral activations were identified in the parietal lobe, specifically in the superior and inferior parietal lobules.

## Discussion

This study presents an fMRI experiment conducted within a virtual rehabilitation environment. The experiment involved a sample of 24 healthy volunteers performing both action execution and action observation tasks, with and without a virtual limb. The findings provide insights that could inform the design of more adaptable and flexible virtual rehabilitation environments, as discussed in the following subsections.

### Whole-brain activations during action execution

As regards the two execution conditions (*dot exe* and *hand exe*), activations were found in cortical and noncortical regions typically involved in the performance of visuomotor tasks (Modroño et al. 2013, 2015, 2020; Turella et al. 2020). Such activated regions were essentially located in the occipital lobe and the somatosensory and motor systems (frontoparietal regions), and were very similar for the dot execution and the hand execution condition, as Fig. 2 shows. In fact, no significant differences were found between the two execution conditions in the motor or the somatosensory systems. The only significant differences associated with the presence of the virtual limb appeared in occipital regions related to the perception of body parts (Astafiev et al. 2004; Downing et al. 2001) that were located in small clusters, and such differences only appeared when uncorrected statistics for multiple comparisons were employed. Therefore, the presence or the absence of the virtual limb during the action execution neurorehabilitation task was not associated with substantial differences in motor or somatosensory activity.

### Whole-brain activations during action observation

In the two observation conditions (*dot obs* and *hand obs*), activations were found in the well-known action observation network (Hardwick et al. 2018), which includes occipital and frontoparietal regions typically involved in observing similar or related visuomotor tasks (Di Cesare et al. 2015; Modroño et al. 2013; Simonelli et al. 2024) and overlap with frontoparietal regions often discussed in the MNS literature. Specifically, during the hand observation condition, participants observed the movements of a virtual arm. The resulting brain activity aligns with previous neuroimaging studies using virtual limbs in their tasks (Adamovich et al. 2009; Prochnow et al. 2013). Similarly, during the dot observation condition, activation in comparable regions was observed even though participants were merely watching the movements of a small virtual object (the dot). When directly comparing the two observation conditions, which differed only in the presence or absence of the virtual limb, no significant differences in brain activity were found at a corrected statistical threshold (using uncorrected statistics, small clusters of increased activity appeared in occipital regions associated with body part perception). The factorial analysis also showed a heterogeneous observation > execution effect (Supplementary Figure S2). Given the exploratory nature of this contrast, we interpret it cautiously, as it may reflect perceptual, attentional, or contextual aspects of stimulus monitoring rather than stronger overall motor-related recruitment during observation itself.

These results suggest that the frontoparietal activity observed during the hand observation condition may not be determined solely by the visual presence of the limb, but also by the observation of actions linked to the observer’s motor repertoire (Modroño et al. 2013). The presence of the red dot in the hand condition may have contributed to associating the dot’s movements with those of the virtual arm. However, this is likely not the sole factor driving frontoparietal activation. Prior research shows that similar activations can occur when observing the movements of virtual objects that are not directly associated with virtual limbs but are perceived as extensions of the observer’s hand (Maravita and Iriki 2004). This raises the question of what would happen in the absence of prior execution runs or practice periods. This scenario could occur in a clinical setting, for instance, with a patient who cannot move their arm at the start of therapy. Since the movements of the virtual limb were physically accurate and plausible, they could be considered part of the participant’s motor repertoire. Consequently, mirror-related activity could still be expected in such cases, as observed in some action observation experiments that do not include practice periods (Plata Bello et al. 2015). In the dot condition, frontoparietal action observation/execution activity could also be expected due to prior experiences with related tasks, such as controlling a cursor on a screen (Modroño et al. 2013).

### Shared activations during action observation and action execution

The conjunction analyses of the execution and observation conditions (Fig. 3) reveal that both task variants produced widespread activations in frontoparietal regions commonly activated during action observation and execution (Cattaneo and Rizzolatti 2009), particularly in dorsal parts. Previous research links these dorsal regions to the observation and execution of transitive movements (those involving object interaction) and reaching actions (Buccino et al. 2001; Di Cesare et al. 2015; Di Dio et al. 2013; Filimon et al. 2007; Modroño et al. 2013). These types of movements were integral to our task, where a virtual effector (arm or dot) reached toward a target. These findings support the notion that activations associated with observing reaching movements are dorsally located within parietofrontal regions often discussed in the MNS literature (Di Dio et al. 2013). This insight can guide the design of tasks and protocols for action observation neurorehabilitation systems. Furthermore, these activated regions are particularly relevant in neurorehabilitation contexts as they are linked to the sensorimotor system, which is often impaired in stroke patients (Boenstrup et al. 2018; Boni et al. 2023; Lv et al. 2022; Robinson-Bert and Woods 2024; Seitz et al. 2024).

### ROI analyses (action observation and action execution)

The ROI analyses were broadly consistent with the whole-brain results in not showing corrected hand–dot differences in the predefined frontoparietal and sensorimotor regions. In execution, the effects were uniformly small, suggesting that the visual presence of a virtual hand did not substantially change ROI-level activation relative to the dot condition. During observation, nominally significant uncorrected effects were observed in bilateral IFG, in the direction of greater activation for the dot condition; however, these did not survive correction for multiple comparisons and should therefore be considered exploratory. Taken together, the ROI analyses do not support stronger frontoparietal or sensorimotor recruitment for the hand condition. Future studies could complement the present univariate and ROI-based analyses with multivariate approaches such as MVPA/RSA to determine whether the hand and dot conditions differ in distributed neural patterns despite their broadly similar mean activation profiles.

### Implications for virtual rehabilitation

It has been found that the neurorehabilitation task (in both its execution and observation variants) was associated with widespread activation in frontoparietal action observation/execution regions and that the presence or absence of a virtual limb was not associated with substantial differences in these activations. These findings may be relevant to action observation-based rehabilitation systems, which involve patients observing motor actions, sometimes followed by executing the same actions (depending on the patient’s motor abilities). Most clinical trials incorporate action execution after observation, gradually increasing task complexity as the patient progresses (Borges et al. 2018). Our results suggest that the desired motor cortex activations, which form the physiological basis for this therapy, could be achieved without requiring a virtual limb’s presence. The present paradigm was deliberately simplified (cancellation-like selection task with a cursor-like effector) in order to control variables and isolate the visual presence of a limb. This simplification limits ecological validity relative to rehabilitation tasks centered on meaningful object-oriented actions. Therefore, our conclusions should be interpreted as applying primarily to VR tasks with simple visuomotor mappings; extension to object-based functional actions requires direct empirical confirmation. By not requiring the visual presence of a virtual limb, system developers gain greater flexibility in designing tasks. For instance, some tasks may be more effective or engaging without a virtual limb, potentially enhancing patient adherence to therapy and promoting motor learning (Dhawale et al. 2017). Additionally, many interventions have repurposed recreational off-the-shelf video games that do not feature visible effector limbs (Bonnechere et al. 2016; Sagary et al. 2023). Based on our results, observing the actions performed in these games—whether or not paired with execution—might engage motor systems in patients, although this remains to be tested in clinical populations. Note that even when execution is not possible, observing video game actions can be highly motivating. Many individuals enjoy watching others play video games through platforms like Twitch (Sjoblom and Hamari 2017), and this observational behavior might benefit neurorehabilitation by engaging motor-related brain activity, as our findings suggest. The present results were obtained from healthy volunteers; however, given that the tasks imposed a low cognitive load, they may be relevant for future studies in stroke patients. For these reasons, it would be worthwhile to pursue this line of research further by conducting additional experiments involving clinical populations.

### Limitations

A design feature that should be considered when interpreting the results is that execution (including brief practice) preceded observation. Therefore, we cannot rule out that part of the observation–execution overlap reflects visuomotor learning and/or prior familiarity with cursor-like control, rather than independence from the visual presence of a virtual limb. A future design with counterbalanced run order or AO-only / AE-only groups would allow these factors to be dissociated more clearly. In addition, because no independent measure of attention (e.g., eye tracking) was available, subtle differences in attentional allocation between the dot and hand displays cannot be completely excluded. Nevertheless, the visual characteristics of the task were such that attention was naturally directed toward the effector and its movement toward the target. These findings were obtained in healthy adults and therefore should not be generalized directly to stroke or other clinical populations. In stroke, the response to action observation and action execution may depend on lesion location and extent, motor impairment severity, the presence of apraxia or neglect, attentional factors, and other clinical variables. Accordingly, we present the current results as a basis for designing and prioritizing future validation studies in clinical populations rather than as an immediate clinical recommendation.

### Conclusions

In this fMRI study of a virtual neurorehabilitation task, action execution and action observation were associated with similar frontoparietal and sensorimotor activations when actions were displayed either with a virtual hand or with a moving dot. Under the present task conditions, the visual presence of a virtual limb did not substantially modify the overall frontoparietal/sensorimotor activations. These findings support the development of more flexible virtual rehabilitation tasks and broaden the range of visual effectors that may be considered in future system design. Further studies in clinical populations will be important to determine the translational relevance of these findings.

**Table 1:** Main anatomical structures activated during the observation and execution conditions

Hemisphere	Region	BA	Cluster size (voxels)	[X Y Z]	Peak T value
DOT OBSERVATION					
L/R	precentral gyrus, supp. motor area, inf. frontal gyrus, parietal lobe, supramarginal, precuneus, hippocampus, occipital lobe, lingual gyrus, fusiform gyrus, calcarine, cuneus, cerebellum, thalamus	6,7,40,18,19,37	51,166	16–90 2	13.91
L	middle temporal gyrus, sup. temporal gyrus, temporal pole	38,20,21	107	-48 8–26	2.70
R	middle frontal gyrus, inf. frontal gyrus	11,47,10	223	40 52 0	3.74
L/R	superior frontal gyrus, medial frontal gyrus	10	175	10 66 10	3.60
R	superior temporal gyrus	22	26	66 − 36 14	2.79
R	precentral gyrus, inf. frontal gyrus	6,44	33	64 4 14	3.03
L	superior frontal gyrus	9,10	69	-16 56 32	2.84
R	cingulate gyrus	24,23	28	6–10 26	2.57
HAND OBSERVATION					
L/R	precentral gyrus, supp. motor area, inf. frontal gyrus, parietal lobe, supramarginal, precuneus, hippocampus, occipital lobe, lingual gyrus, fusiform gyrus, calcarine, cuneus, cerebellum, thalamus	6,7,40,18,19,37	28,963	-6 -90 -10	16.3
L	middle frontal gyrus, inf. frontal gyrus	10	64	-32 54 0	3.48
L	precentral gyrus, supp. motor area, inf. frontal gyrus	6	3327	-42 -4 44	6.6
R	precentral gyrus, inf. frontal gyrus	6,9	62	48 2 40	2.93
L	superior frontal gyrus	9,8	29	-16 40 48	2.76
R	precentral gyrus	6	323	28 0 50	4.2
DOT EXECUTION					
L/R	left postcentral gyrus, precentral gyrus, supp. motor area, inf. frontal gyrus, parietal lobe, supramarginal, precuneus, hippocampus, occipital lobe, lingual gyrus, fusiform gyrus, calcarine, cuneus, cerebellum, thalamus, caudate, putamen	3,4,6,7,18,19,37,40	59,332	-38 -24 58	12.83
R	frontal lobe		50	18 32 2	2.81
R	putamen, insula	13	113	22 14 2	2.62
L	frontal lobe		34	-20 32 4	2.47
L	middle frontal gyrus, inf. frontal gyrus	10,46,9	106	-42 40 26	3.29
HAND EXECUTION					
L/R	left postcentral gyrus, precentral gyrus, supp. motor area, inf. frontal gyrus, parietal lobe, supramarginal, precuneus, hippocampus, occipital lobe, lingual gyrus, fusiform gyrus, calcarine, cuneus, cerebellum, thalamus, caudate, putamen	3,4,6,7,18,19,37,40	72,637	-34 -74 -18	14.16
R	middle frontal gyrus	10	61	36 56 − 2	3.33
L	medial frontal gyrus	10	44	-12 66 − 2	3.14
R	middle frontal gyrus	46,10	26	50 38 20	2.72
L	superior frontal gyrus		26	-14 60 22	2.29
L	middle frontal gyrus, inf. frontal gyrus	9,10,46	188	-40 40 26	3.74
DOT OBSERVATION > HAND OBSERVATION: No significant activation (even with uncorrected statistics*)					
HAND OBSERVATION > DOT OBSERVATION: Only significant activations when using uncorrected statistics*					
L	middle occipital gyrus, inferior temporal gyrus	37,19	15	-48 -74 -4	3.59
L	occipital lobe, calcarine sulcus	17	15	-6 -90 -8	3.38
DOT EXECUTION > HAND EXECUTION: No significant activation (even with uncorrected statistics*)					
HAND EXECUTION > DOT EXECUTION: Only significant activations when using uncorrected statistics*					
L	middle occipital gyrus, inferior temporal gyrus	37,19,18	244	-50 -74 -2	4.59

Anatomical structures and Brodmann Areas (BA) are shown with corresponding MNI coordinates of peak activity in each cluster. Threshold: *p* < 0.05 FDR corrected at the voxel level, k = 25. *Threshold for the uncorrected results: *p* < 0.001, k = 10.

## Supplementary Information

Below is the link to the electronic supplementary material.

Supplementary Material 1

Supplementary Material 2

## Data Availability

The data that support the findings of this study are available from the corresponding author upon request.
