# Supplementary material for: Similar Sensorimotor Activations with and without Virtual Limbs During Action Execution and Observation in Neurorehabilitation Systems
Source: Brain Topogr. 2026 May 27;39(4):61. doi: 10.1007/s10548-026-01219-1 (PMC13216155; doi:10.1007/s10548-026-01219-1)
Supplement: Supplementary file 1 — Supplementary Material 1 [file 10548_2026_1219_MOESM1_ESM.docx]

**Supplementary Table 1.** A priori ROI comparisons between hand and dot conditions during execution and observation. Values are mean within-ROI differences (hand − dot). Positive values indicate greater activation in the hand condition; negative values indicate greater activation in the dot condition. None of the comparisons survived correction for multiple comparisons (Bonferroni).

**Execution**

| ROI | Hand − Dot | 95% CI | Cohen’s dz | p (unc.) | p (corr.) |
| --- | --- | --- | --- | --- | --- |
| L IPL (BA40) | 0.043 | [-0.122, 0.209] | 0.11 | 0.593 | ns |
| L IFG (BA44/45) | 0.040 | [-0.125, 0.205] | 0.10 | 0.624 | ns |
| L M1 (BA4) | -0.018 | [-0.148, 0.112] | -0.06 | 0.776 | ns |
| L Premotor (BA6) | 0.017 | [-0.113, 0.148] | 0.06 | 0.786 | ns |
| L SPL (BA7) | 0.083 | [-0.093, 0.258] | 0.20 | 0.339 | ns |
| L SMA | 0.013 | [-0.125, 0.150] | 0.04 | 0.851 | ns |
| R IPL (BA40) | 0.002 | [-0.127, 0.131] | 0.01 | 0.979 | ns |
| R IFG (BA44/45) | 0.036 | [-0.091, 0.163] | 0.12 | 0.563 | ns |
| R M1 (BA4) | -0.009 | [-0.119, 0.100] | -0.04 | 0.859 | ns |
| R Premotor (BA6) | 0.003 | [-0.115, 0.121] | 0.01 | 0.958 | ns |
| R SPL (BA7) | 0.020 | [-0.160, 0.199] | 0.05 | 0.822 | ns |
| R SMA | 0.002 | [-0.122, 0.127] | 0.01 | 0.969 | ns |

**Observation**

| ROI | Hand − Dot | 95% CI | Cohen’s dz | p (unc.) | p (corr.) |
| --- | --- | --- | --- | --- | --- |
| L IPL (BA40) | -0.044 | [-0.157, 0.070] | -0.16 | 0.435 | ns |
| L IFG (BA44/45) | -0.142 | [-0.263, -0.021] | -0.49 | 0.024 | ns |
| L M1 (BA4) | -0.046 | [-0.168, 0.076] | -0.16 | 0.446 | ns |
| L Premotor (BA6) | -0.057 | [-0.166, 0.053] | -0.22 | 0.297 | ns |
| L SPL (BA7) | -0.048 | [-0.208, 0.111] | -0.13 | 0.538 | ns |
| L SMA | -0.043 | [-0.171, 0.086] | -0.14 | 0.498 | ns |
| R IPL (BA40) | -0.151 | [-0.325, 0.024] | -0.37 | 0.087 | ns |
| R IFG (BA44/45) | -0.133 | [-0.265, -0.001] | -0.42 | 0.049 | ns |
| R M1 (BA4) | -0.081 | [-0.249, 0.088] | -0.20 | 0.333 | ns |
| R Premotor (BA6) | -0.090 | [-0.198, 0.019] | -0.35 | 0.100 | ns |
| R SPL (BA7) | -0.049 | [-0.238, 0.140] | -0.11 | 0.596 | ns |
| R SMA | -0.101 | [-0.256, 0.054] | -0.28 | 0.190 | ns |
