# Supplementary material for: Similar Sensorimotor Activations with and without Virtual Limbs During Action Execution and Observation in Neurorehabilitation Systems
Source: Brain Topogr. 2026 May 27;39(4):61. doi: 10.1007/s10548-026-01219-1 (PMC13216155; doi:10.1007/s10548-026-01219-1)
Supplement: Supplementary file 2 — Supplementary Material 2 [file 10548_2026_1219_MOESM2_ESM.docx]

**SUPPLEMENTARY FIGURES.** These supplementary figures present the whole-brain results of the 2 × 2 factorial analysis, with Effector (dot, hand) and Run (execution, observation) as factors. They illustrate the main effects of Effector and Run, as well as the Effector × Run interaction. Results are shown at an exploratory threshold of p < 0.001 uncorrected, k = 5 voxels.


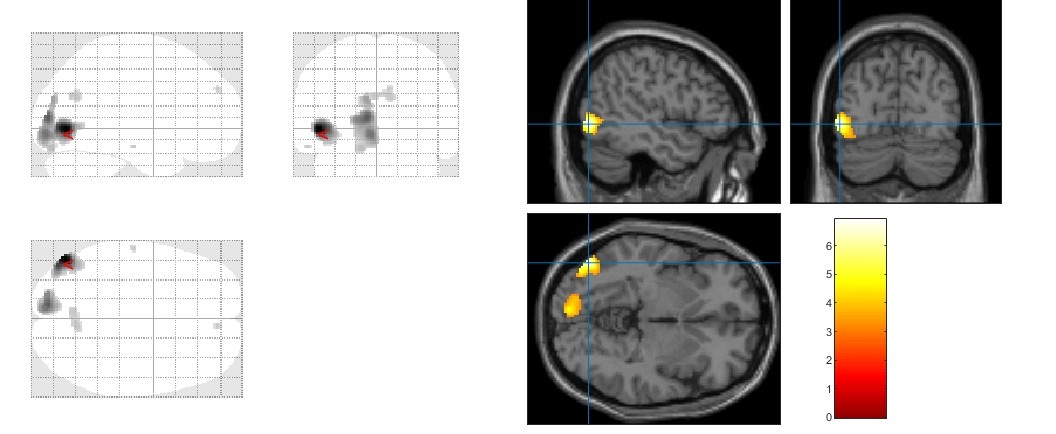


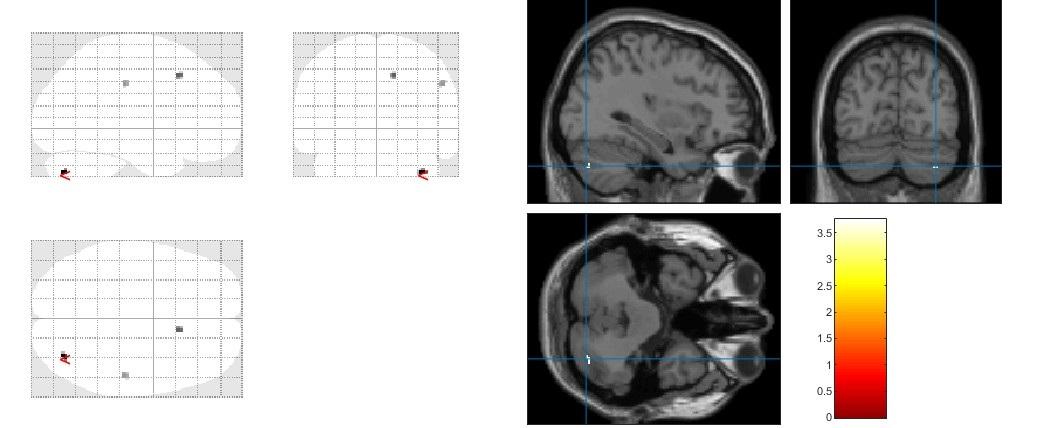


**Supplementary Figure S1.** **Main effect of Effector** in the 2 × 2 factorial design. Whole-brain maps showing (TOP) Hand > Dot and (BOTTOM) Dot > Hand.


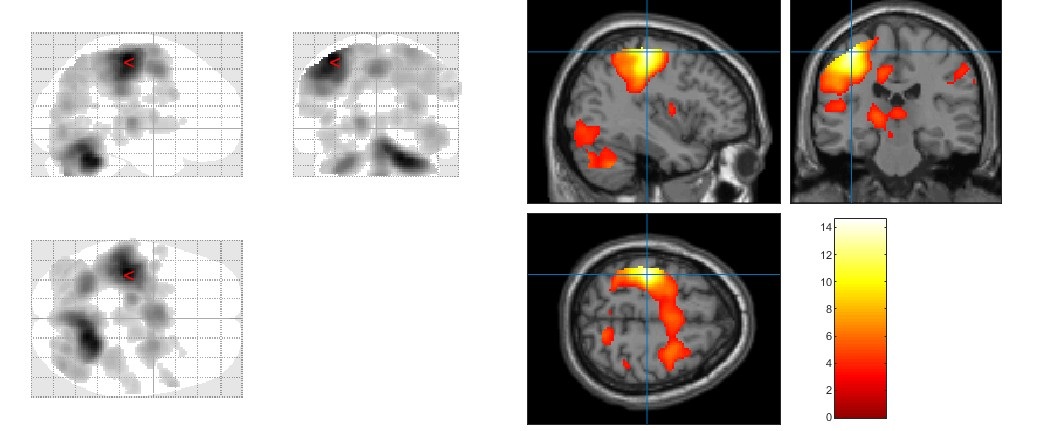


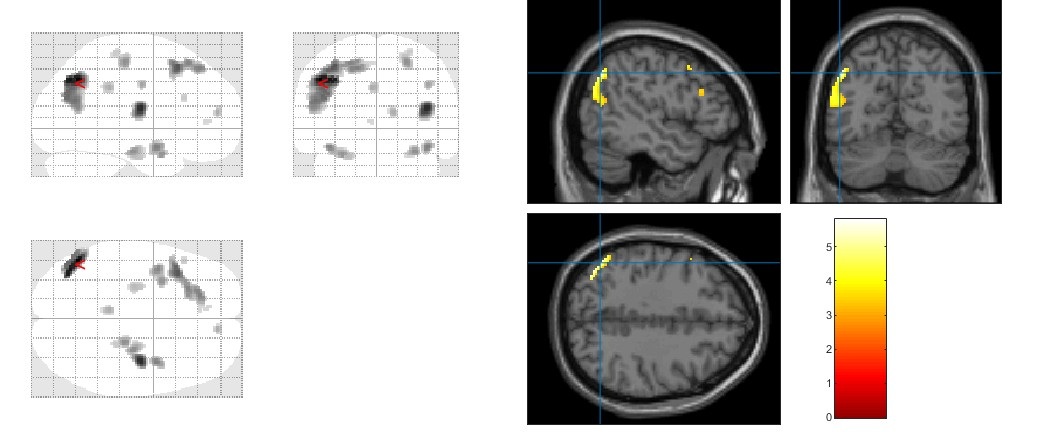


**Supplementary Figure S2.** **Main effect of Run** in the 2 × 2 factorial design. Whole-brain maps showing (TOP) Execution > Observation and (BOTTOM) Observation > Execution.


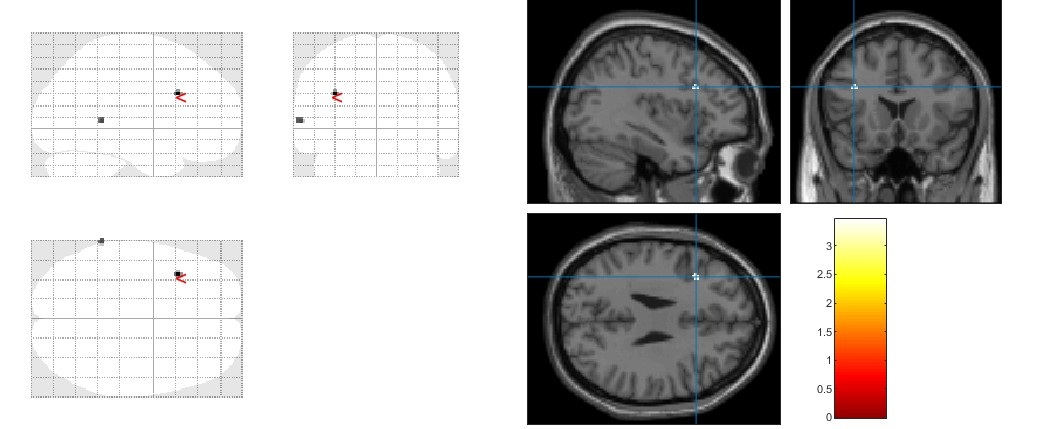


**Supplementary Figure S3. Effector × Run interaction** in the 2 × 2 factorial design. Whole-brain maps showing an interaction contrast. No suprathreshold activation was observed for the inverse interaction contrast.
